# Supplementary material for: A high-resolution mRNA expression time course of embryonic development in zebrafish
Source: eLife. 2017 Nov 16;6:e30860. doi: 10.7554/eLife.30860 (PMC5690287; doi:10.7554/eLife.30860)
Supplement: Supplementary file 6. [file elife-30860-supp6.zip › biolayout-clusters-files/Cluster131-genes.html]

Cluster131


# Cluster131: Genes

| Ensembl ID | Gene Name | Chr | Start | End | Biotype |
| --- | --- | --- | --- | --- | --- |
| ENSDARG00000003041 | CABZ01111496.1 | 20 | 54370474 | 54385783 | protein\_coding |
| ENSDARG00000089607 | CU929037.1 | 17 | 49466616 | 49468012 | protein\_coding |
| ENSDARG00000058806 | apoa1bp | 19 | 7489310 | 7493722 | protein\_coding |
| ENSDARG00000023903 | atf2 | 9 | 2346408 | 2400071 | protein\_coding |
| ENSDARG00000003872 | ccdc102a | 7 | 28453725 | 28566168 | protein\_coding |
| ENSDARG00000076076 | crtc1b | 11 | 6893107 | 6948203 | protein\_coding |
| ENSDARG00000024775 | si:ch211-129c21.1 | 22 | 22291748 | 22315783 | protein\_coding |
| ENSDARG00000069168 | si:dkey-73n10.1 | 18 | 18942418 | 18953032 | protein\_coding |
| ENSDARG00000074531 | spag9a | 3 | 18153468 | 18223883 | protein\_coding |
| ENSDARG00000024540 | tspan36 | 8 | 29717388 | 29725719 | protein\_coding |
